# A Rare Case of Large-Vessel Vasculitis following Checkpoint Inhibitor Therapy and Pegfilgrastim

**DOI:** 10.1155/2022/7295305

**Published:** 2022-02-23

**Authors:** Joseph Mort, Shipra Maheshwari, Nayanika Basu, Patrick Dillon, Kevin Brady, Harry Bear, Trish Millard

**Affiliations:** ^1^Department of Medicine, University of Virginia Health System, 1215 Lee Street, Charlottesville, VA 22908, USA; ^2^Emily Couric Cancer Center, University of Virginia Health System, 1240 Lee Street, Charlottesville, VA 22903, USA; ^3^Massey Cancer Center and Department of Surgery, Virginia Commonwealth University and VCU Health, Box 980011, Richmond, VA 23298-0011, USA

## Abstract

Checkpoint inhibitors (CPIs) and pegfilgrastim, a long-acting growth factor agent, are vital components of current cancer treatments. Immune-related adverse events (irAEs) such as colitis and pneumonitis are well-established toxicities associated with CPI therapy. However, large-vessel vasculitis secondary to CPI utilization is reported only in rare case reports and case series. Interestingly, large-vessel vasculitis has also been reported as a rare complication of pegfilgrastim use. We present a 59-year-old female with left stage IIA (cT2N0M0) triple-negative breast cancer receiving neoadjuvant decitabine and pembrolizumab prior to neoadjuvant chemotherapy (NAC). NAC included standard-of-care dose dense doxorubicin and cyclophosphamide (ddAC) supported with pegfilgrastim use followed by weekly carboplatin and paclitaxel. After receiving her second cycle of ddAC with pegfilgrastim, the patient reported five days of left shoulder and arm pain. Subsequent CT imaging demonstrated wall thickening and inflammatory changes surrounding the left subclavian artery, aortic arch, left carotid artery, proximal innominate arteries, and the mid internal carotid arteries and its branching vessels. These findings were extremely concerning for large-vessel vasculitis. Excluding CPI therapy and pegfilgrastim use, no additional inciting event or medication that the patient was exposed to was noted to be associated with large-vessel vasculitis. We present this case to report on this rare but severe complication from commonly utilized agents in cancer treatment. We also extend the possibility of large-vessel vasculitis development in relation to the COVID-19 vaccine due to shared ingredients found in both the vaccine and pegfilgrastim. It is important to outline the treatment used for such a complication as no standardized treatment has been established for large-vessel vasculitis caused by CPI therapy or pegfilgrastim use.

## 1. Introduction

CPIs as a component of cancer treatment have revolutionized patient outcomes. However, these medications must be used with caution as they carry substantial risk for causing autoimmune side effects or irAEs because of stimulation of the immune system. IrAEs have been reported to affect nearly every organ system in the body, most frequently affecting the gastrointestinal, hepatic, pulmonary, cutaneous, and endocrine systems [1, 2]. The most frequent rheumatologic irAEs that occur due to CPI therapy are arthralgias and myalgias (11.0% and 7.3%, respectively) [3]. Vasculitis is a rare rheumatologic irAE of CPI therapy that has only been specifically described in case reports and case series [4]. Of the CPI-vasculitis cases reported in the literature, large-vessel vasculitis is the most common subtype [5].

Cases of large-vessel vasculitis induced by granulocyte colony-stimulating factors (G-CSFs) have also been reported [6–13]. In a large observational study of 102,014 subjects with malignant neoplasms in Japan, 0.47% of subjects treated with G-CSF developed aortitis compared to only 0.01% in subjects who did not receive G-CSF [9]. Although exposure to either pegylated or nonpegylated G-CSF was evaluated in this study, more patients developed aortitis after receiving pegylated G-CSF utilization [6–9]. Pegfilgrastim is a form of G-CSF with an attached polyethylene glycol (PEG) moiety that extends duration of action to allow for less frequent administrations compared to nonpegylated G-CSF which is injected daily. Pegylated G-CSF has demonstrated greater efficacy in prevention of severe neutropenia, dose reductions, and treatment delays when administering dose-dense chemotherapy in breast cancer [14, 15]. However, the addition of this PEG moiety could theoretically lead to an immunostimulatory effect causing the immune cells to target native vessels in the body. In one case of large-vessel vasculitis after pegfilgrastim administration, the patient was transitioned to nonpegylated G-CSF with additional cycles of chemotherapy without relapse of vasculitis [16].

Given the rare nature of CPI and G-CSF-induced vasculitis, standardized treatment of this adverse effect has not yet been established. Herein, we present a rare case of large-vessel vasculitis possibly induced by either CPI or G-CSF and the treatment that followed resulting in rapid resolution of symptoms.

## 2. Case Presentation

**Figure 1:**
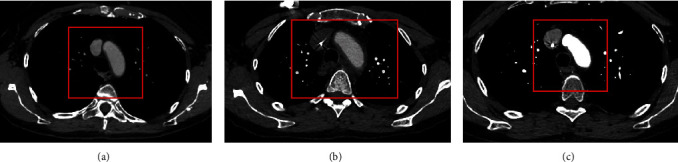
(a) CT chest with contrast of the patient on 2/11/21. (b) CT chest with contrast of the patient on 5/03/21. (c) CT chest with contrast of the patient on 7/01/21. Between February and March 2021, this patient received both pembrolizumab and pegfilgrastim. Her May CT was significant for wall thickening and inflammatory changes surrounding the left subclavian artery extending into the aortic arch with involvement of the left carotid artery and proximal innominate arteries, new findings as compared to her February CT. After the initiation of a long steroid taper in May, the patient's CT chest in July showed marked improvement of the wall thickening and inflammatory changes of the aortic arch and arch vessels, most compatible with resolving vasculitis.

A 59-year-old female with left stage IIA (cT2N0M0) triple-negative breast cancer was treated on a clinical trial with neoadjuvant chemoimmunotherapy [17]. The investigational portion of treatment included decitabine (4 doses) administered on days 1–5 and pembrolizumab administered on days 8 and 22, prior to starting neoadjuvant chemotherapy (investigational use of an FDA approved agent). The patient then received standard-of-care chemotherapy with four cycles of ddAC and pegfilgrastim support followed by twelve weekly doses of carboplatin and paclitaxel. After receiving her second cycle of ddAC with pegfilgrastim, the patient reported 5 days of worsening left shoulder and arm pain. The pain started in the left scapula and radiated down the left arm to the back and under the left breast. It was pleuritic and severe in nature. The pain worsened with active but not passive range of motion. She denied chest pain, dyspnea, or fevers but also reported a pruritic, papular rash over her chest and back that started two weeks before the onset of arm pain. An X-ray of the left humerus showed no fracture. Given the pleuritic nature of her pain, the patient underwent a contrasted chest CT to assess for pulmonary embolism. This showed wall thickening and inflammatory changes surrounding the left subclavian artery extending into the aortic arch with involvement of the left carotid artery and proximal innominate arteries (Figure 1). These imaging findings were concerning for large-vessel vasculitis, and the patient was admitted to the hospital for work up and management.

Vital signs were within normal limits. Basic labs revealed a neutrophilic predominant leukocytosis and mild normocytic anemia. Her kidney function was normal and urinalysis showed trace protein and blood; the urine protein-to-creatinine ratio was mildly elevated at 0.21. Liver function tests were within normal limits. Her troponin and BNP were in the normal ranges. ECG on admission was normal and a transthoracic echocardiogram showed a normal ejection fraction, cavity size, and wall thickness. Inflammatory markers revealed a normal sedimentation rate, but C-reactive protein was elevated to 847 mmol/L (normal < 48 mmol/L). Rheumatology was consulted to assist with the work up of her large-vessel vasculitis. Serologic testing demonstrated a negative antinuclear antibody, rheumatoid factor, myeloperoxidase antibody, proteinase 3 antibody, and normal C4 complement level. Hepatitis serologies revealed a nonreactive hepatitis C antibody and evidence of prior immunization to hepatitis B. Creatine kinase was ordered to rule out myositis and was normal. Blood cultures were obtained to assess for infection and no microbial growth was identified. Further vascular imaging was acquired to assess for involvement of other organs. CT of the head and neck with contrast revealed that the wall thickening of the common carotid arteries extended cranially to the level of the midinternal carotid arteries but there was no significant stenosis or involvement of the intracranial vessels. The vessels in the abdomen and pelvis were without inflammatory changes.

After the above workup, the differential diagnosis for this patient's large-vessel vasculitis included CPI-induced vasculitis due to pembrolizumab- or pegfilgrastim-induced vasculitis. As part of the clinical trial for triple-negative breast cancer, she received two doses of pembrolizumab 45 and 31 days prior to onset of her left arm pain. Two doses of pegfilgrastim with ddAC were given 22 and 8 days prior to onset of her symptoms. Of note, she also received the two-dose Pfizer COVID-19 vaccine series prior to starting therapy for her breast cancer with doses administered two and three months prior to symptom onset. After being started on systemic steroids (prednisone 1 mg/kg) for treatment in the hospital, the patient demonstrated rapid improvement in her symptoms. The patient was discharged with plans to continue on a slow steroid taper.

She had continued improvement in her left arm pain and reported only mild residual weakness at follow-up 2 weeks after discharge. She did note a change in the character of her vision and was referred to ophthalmology for further evaluation with no findings of ocular inflammation seen on eye exam. Repeat inflammatory markers two weeks after discharge were normal, and repeat imaging 8 weeks later showed marked improvement in vasculitis (Figure 1). She completed a slow steroid taper over 12 weeks with no recurrence of symptoms.

The patient did not receive additional immunotherapy after onset of symptoms as she had already completed all doses per the clinical trial. The frequency of subsequent chemotherapy doses was decreased and carboplatin eventually discontinued to allow for avoidance of additional G-CSF doses and prevention of large-vessel vasculitis recurrence.

## 3. Discussion

While CPIs have significantly improved outcomes for patients with a variety of cancers, management of the associated irAEs requires prompt treatment and close monitoring by the treatment team. One rare irAE is vasculitis. One series of 20 cases of CPI-induced vasculitis revealed that large-vessel vasculitis (6 cases) was the most commonly reported type of vasculitis but medium vessel, small vessel, and single-organ vasculitis were also observed. Cases of large-vessel vasculitis included giant cell arteritis (GCA) and aortitis/periaortitis. Median time to onset after CPI therapy was 3 months but ranged from 1.2 to 6 months [5]. The case of large-vessel vasculitis we present above occurred after 2 doses of pembrolizumab that were given one and a half and one month prior to onset of her symptoms. Although this is a shorter duration of symptom onset after CPI therapy than the median onset of 3 months, it still lies within the reported range. Involvement of the aortic arch and major branches fits with the previously reported location of vessel involvement. It was also important to investigate irAEs involving other organ systems. The patient received a thorough workup to rule out concomitant organ involvement. Of note, special care was devoted to ensuring that the patient had no symptoms of GCA given its high prevalence in CPI-vasculitis and she was referred to ophthalmology in the outpatient setting after discharge.

In this case, it was imperative to consider all possible etiologies of vasculitis. Although this patient's case fits with CPI-induced vasculitis, cases of G-CSF-induced large-vessel vasculitis and aortitis have also been reported [6–13]. G-CSF aids the differentiation and maturation of neutrophils as well as stimulates inflammatory cytokine production. For these reasons, G-CSF may trigger vasculitis [11]. Interestingly, one case report suggests a higher incidence of G-CSF-related vasculitis in advanced cancer compared to earlier stage disease [11]. Our patient received pegfilgrastim with her first two cycles of ddAC 22 and 8 days prior to onset of her symptoms. The time to onset of symptoms after administration of G-CSF was 1–13 days in a review of seventeen cases and in a separate review of 5 cases in Japan as well as median onset of 8 days in a case series of 6 patients in Finland with breast cancer who developed large-vessel vasculitis [6, 7, 12]. The timeline of development of our patient's large-vessel vasculitis fits with the timeline in the cases previously reported. As mentioned above, pegfilgrastim was more commonly associated with large-vessel vasculitis compared to nonpegylated G-CSF [6–9]. In one case of large-vessel vasculitis after pegfilgrastim administration, the patient was transitioned to nonpegylated G-CSF with additional cycles of chemotherapy administered without relapse of vasculitis [16]. Still, other case reports indicate that both pegylated and nonpegylated G-CSF can be associated with the development of aortitis [10]. One case report suggested that those who received pegylated G-CSF required steroids and those who received nonpegylated G-CSF had resolution of vasculitis without steroids [13]. Given the potential for recurrent vasculitis with either pegylated or nonpegylated G-CSF, as well as patient's general chemotherapy tolerance to date, our patient's ddAC was switched to every three weeks of dosing so that growth factor support was no longer preemptively required; her symptoms did not recur. She was also treated with a prolonged steroid taper as would be typical for irAE from CPI. It should be noted that there is limited available data on the safety of CPI given with G-CSF; thus, it is hypothesized that exposure to both agents may increase one's risk for vasculitis.

Other possible contributing factors in this case were the patient's COVID-19 vaccination and her chemotherapy. Two months prior to starting chemotherapy, our patient received the mRNA COVID-19 vaccine. At the time of this patient's presentation, there were no reported cases of vasculitis secondary to the COVID-19 vaccine but both of the mRNA vaccines (Pfizer-BioNTech and Moderna) contain PEG as an ingredient [18]. Although it is unclear to what degree (if any) vaccination contributed to this patient's presentation, it is possible that immune stimulation associated with the vaccine in the presence of PEG made the patient more sensitive to subsequent exposure to PEG in the form of pegfilgrastim when she initiated chemotherapy. It will be important to monitor for additional cases of vasculitis associated with pegfilgrastim as more individuals receive the COVID-19 vaccine. The patient's prior dose of decitabine was also queried as a contributory factor, but the experience with this agent and the timeline suggest against it playing a role in vasculitis.

Since both CPI-vasculitis and pegfilgrastim-/G-CSF-induced vasculitis are rare conditions, standardized treatment does not exist. However, in the cases of CPI-vasculitis reported, discontinuation of the CPI therapy and initiation of high-dose glucocorticoids resulted in resolution of symptoms. Glucocorticoid regimens included high-dose oral prednisone (1–2 mg/kg daily) or several days of IV methylprednisolone followed by slow taper of oral glucocorticoids [5]. Disease-modifying antirheumatic drugs have been used to treat rheumatologic irAEs in patients with symptoms refractory to glucocorticoids or as steroid-sparing agents, including in two cases of CPI-vasculitis [3, 19, 20]. Use of tumor necrosis factor inhibitors (predominantly infliximab) and the interleukin-6 inhibitor tocilizumab has also been used with success for rheumatologic irAEs, but to our knowledge, they have not been used for CPI-vasculitis [3]. Our patient's symptoms and imaging findings are resolved with high-dose oral prednisone and a subsequent prolonged taper along with stopping pegfilgrastim. Her CPI therapy had previously been completed prior to onset of symptoms. Some cases of spontaneous resolution of symptoms and imaging findings of G-CSF vasculitis without steroid treatment have been reported. Hoshina and Takei reported 10 cases of G-CSF vasculitis and state that there was no difference in time to remission of aortitis with or without steroids [11]. Yukawa et al. reported 5 cases and differentiated treatment between pegylated G-CSF vasculitis and nonpegylated G-CSF vasculitis, stating that the patients who received pegylated G-CSF required steroids for resolution while those treated with nonpegylated G-CSF did not require steroids [13]. In cases such as these, repeat exposure to CPI and/or growth factors such as G-CSF requires careful assessment of risk to benefit ratio and open discussion with the patient on the uncertainty of safety with repeat exposure.

## 4. Conclusion

The case of therapy-associated large-vessel vasculitis that we present is unique in that it contains several possible etiologies: CPI-induced vasculitis, pegfilgrastim/G-CSF induced vasculitis, or a combination with a potential immunostimulatory contribution of the PEG-containing mRNA COVID-19 vaccine. Although large-vessel vasculitis is a rare adverse event in both of these lifesaving cancer therapies and should not preclude providers from utilizing them in the care of patients, oncologists should consider large-vessel vasculitis as a potential complication of immunotherapy and pegfilgrastim going forward with particular attention to when these medications are used in combination. They should be aware of such a complication when evaluating patients who have received the above agents and present with symptoms of unclear etiology.

## Data Availability

No data was used in the preparation of this case report.
